# Factors Regulating Early Life History Dispersal of Atlantic Cod (*Gadus morhua*) from Coastal Newfoundland

**DOI:** 10.1371/journal.pone.0075889

**Published:** 2013-09-18

**Authors:** Ryan R. E. Stanley, Brad deYoung, Paul V. R. Snelgrove, Robert S. Gregory

**Affiliations:** 1 Ocean Sciences Centre and Biology Department, Memorial University of Newfoundland, St. John's, Newfoundland, Canada; 2 Department of Physics and Physical Oceanography, Memorial University of Newfoundland, St. John's, Newfoundland, Canada; 3 Ecological Sciences Section and Centre of Expertise for Aquatic Habitat Research, Fisheries and Oceans Canada, St. John's, Newfoundland, Canada; University of Connecticut, United States of America

## Abstract

To understand coastal dispersal dynamics of Atlantic cod (*Gadus morhua*), we examined spatiotemporal egg and larval abundance patterns in coastal Newfoundland. In recent decades, Smith Sound, Trinity Bay has supported the largest known overwintering spawning aggregation of Atlantic cod in the region. We estimated spawning and dispersal characteristics for the Smith Sound-Trinity Bay system by fitting ichthyoplankton abundance data to environmentally-driven, simplified box models. Results show protracted spawning, with sharply increased egg production in early July, and limited dispersal from the Sound. The model for the entire spawning season indicates egg export from Smith Sound is 13%•day^−1^ with a net mortality of 27%•day^–1^. Eggs and larvae are consistently found in western Trinity Bay with little advection from the system. These patterns mirror particle tracking models that suggest residence times of 10–20 days, and circulation models indicating local gyres in Trinity Bay that act in concert with upwelling dynamics to retain eggs and larvae. Our results are among the first quantitative dispersal estimates from Smith Sound, linking this spawning stock to the adjacent coastal waters. These results illustrate the biophysical interplay regulating dispersal and connectivity originating from inshore spawning of coastal northwest Atlantic.

## Introduction

The early life history of marine species has long been considered a critical component of recruitment and population structure [1], [2]. Population persistence requires that births and immigration equal, or exceed, deaths and emigration. Therefore, the description of population movement, immigration and emigration, or dispersal is critical to understanding population dynamics [3]. When dispersal is successful, connections among populations are established, the degree of which is defined as connectivity [2].

The egg and larval stages are typically the primary periods for dispersal and consequently have attracted considerable attention for understanding population dynamics [4] often with the goal of improving fisheries management [5]. Transition from the dispersive egg and larval stages to appropriate settlement habitat defines recruitment (immigration) to a location or population. Successful recruitment at this stage is a product of placing propagules into optimal survival conditions [6], coupled with oceanographic conditions that associate settling stage larvae with suitable settlement habitat [7]. For coastal environments, multiple studies attribute strong survival from dispersive egg and larval stages through settlement to local retention [8].

The importance of inshore spawning in coastal Newfoundland has been shown for several species, but especially for Atlantic cod (*Gadus morhua*); coastal embayments offer abundant food resources [9], important spawning sites [4], and myriad habitats suitable for settlement and survival of vulnerable juvenile stages [10]. The heterogeneous mosaic of sub-populations within and between bays in coastal Newfoundland [11] is maintained through migration, spawning and dispersal [12]. Egg and larval dispersive stages of a population can connect spatially extant population sub-units [13], contributing to population structure [14].

In the mid-1990's the discovery of an anomalously large, previously undocumented, spawning aggregation of Atlantic cod in Smith Sound Trinity Bay, Newfoundland [15], [16] drew attention to the potential role of inshore spawning in the recovery of depleted cod stocks. The Smith Sound aggregation represented the largest known cod stock component remaining through the 1990's. The role of this large spawning aggregation in potential recovery of stocks at local or large scales drew considerable attention [12], [15], [16]. Although previous researchers explored topics such as genetic isolation [12], recruitment success [16], reproductive biology [17], and spawning characteristics [18], [19] in the region, no study has detailed early life history dispersal originating in, and propagating away from Smith Sound. The dispersal characteristics of eggs and larvae leaving the Sound remain unresolved, and by closing this gap we begin to address how this large spawning aggregation connects at various spatial scales.

Smith Sound is a relatively small (∼36 km^2^) fjord, bounded by land on three sides, resulting in a natural system in which eggs and larvae disperse from a relatively localized and large source [16], providing a model system to measure marine dispersal from a fairly discrete and known point of origin. This study addresses two broad themes describing dispersal and connectivity of the Smith Sound-Trinity Bay model system: 1) What are the dispersal characteristics of eggs and larvae spawned from a small coastal embayment and how do they vary seasonally? 2) How does movement of eggs and larvae from that small embayment influence subsequent transport in the adjacent bay and beyond? These questions are addressed by describing flow conditions in Trinity Bay and by determining dispersal from empirical data fitted to several biophysical model scenarios using simplified box models to represent the Smith Sound-Trinity Bay system. The results illustrate dispersal potential from Smith Sound, providing new insight into the source dynamics of the Trinity Bay-Smith Sound aggregation. This study builds on current understanding of the biophysical interplay that regulates dispersal, recruitment and therefore connectivity of coastal spawning fish stocks.

## Methods

### Study Area

**Figure 1:**
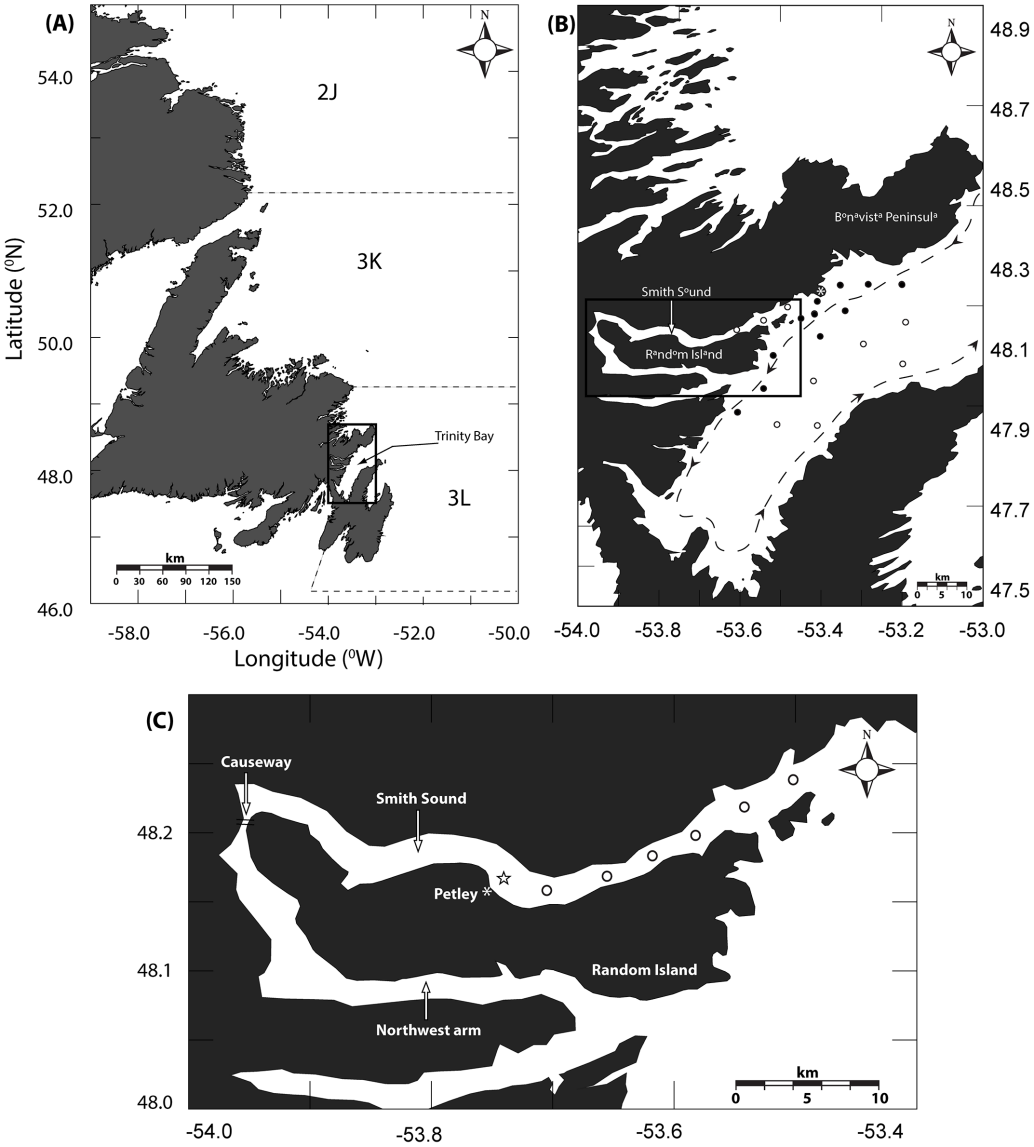
Maps of study area and survey locations depicting. (**a**) Study site relative to NAFO divisions 2J+3KL in the northwest Atlantic Ocean. (**b**) Trinity Bay tucker trawl survey array. Open circles represent eastern Trinity Bay Stations, dashed line represents mean passive flow conditions (Tittensor 2001; 2002), and * represents Bonaventure Head. (**c**) Smith Sound ring net survey stations. Star refers to temperature logger mooring location.

Trinity Bay (48° 2′N, 53° 25′W) is a coastal embayment on the northeast coast of Newfoundland (Fig. 1a,b). The Bay is roughly 100 km long and 30 km wide. A trench runs centrally down the lengthwise axis of the bay with maximum depths exceeding 600 m ending in a sill at the mouth of the bay with a maximum depth ∼ 250 m [20]. Circulation modelling by Yao [21], Davidson et al. [22], and Tittensor et al. [23] reveals a weak anti-cyclonic circulation and the strong influence on circulation by wind forcing, both local and non-local [24]. Smith Sound is a narrow, ∼20 km long by 2 km wide fjord on the western side of Trinity Bay adjacent to Random Island. Trenches running down the center of the Sound extend to depths of 200–350 m and the sill rises to <50 m. A shoal and causeway separate Smith Sound from the adjacent Northwest Arm, restricting the movement of water through a channel that is less than 10 m wide and 2 m deep (Fig. 1c).

### Biological sampling

To estimate temporal characteristics of egg release by spawning cod, ichthyoplankton were sampled bi-weekly at six stations in Smith Sound (Fig. 1c) from March to August in 2006 and 2007 with a 1-m diameter by 3-m long ring net fitted with 333 µm mesh and a General Oceanic flow meter to estimate sample volume. Ring nets were towed at the surface at approximately 4 km•h^−1^ for 20 minutes. Synoptic ichthyoplankton surveys of Trinity Bay provided a basis to infer a dispersal trajectory and the spatial pattern of propagules produced by spawning events in Smith Sound. During the spring (May of 2004 and 2006) and summer (July 2004), ichthyoplankton were collected on board the *CCGS Shamook* over a grid of twenty stations radiating in a “bulls eye” pattern from Smith Sound (Fig. 1b). Double oblique tows were carried out using a 4 m^2^ Tucker trawl fitted with decreasing mesh sizes of 1000, 570, and 333 µm. The trawl was lowered to a maximum depth of 40 m and towed at 4 km•h^−1^ for 20 minutes; 40 m is the approximate depth of the upper mixed layer and has been used a bench mark depth for integrated cod egg and larval sampling in this region [25], [26]. Volumes sampled were estimated using flow meters fitted at the mouth of the trawl. All necessary permits for collecting ichthyoplankton were obtained prior to sampling in accordance with the Canadian Council of Animal Care guidelines. No specific locational permits were required for ichthyoplankton sampling in Smith Sound-Trinity Bay, and no threatened or endangered species were at risk of incidental capture.

Ichthyoplankton samples were preserved in 4% formalin in buffered sea water. In the laboratory, all fish eggs and larvae were removed and identified except where egg stage and larval abundances for taxon exceeded 300 individuals, in which case they were sub-sampled using a Motodo plankton splitter. Eggs of all species were grouped into four taxonomic development stages adapted from methods outlined in Markle and Frost [27]. Samples from May 2006 were also processed for zooplankton abundances [28] following sub-sampling protocols outlined for the cod eggs according to the lowest taxonomic level identifiable. All eggs identified as cod were classified as CHW, representing cod, haddock (*Melanogrammus aeglefinus*) or witch flounder (*Glyptocephalus cynoglossus*). Eggs classified as CHW are indistinguishable to species level until the final developmental stage. In all cases final stage eggs were identified to species [27]. The vast proportion of the CHW eggs was, in fact, cod. Relatively low levels of both witch flounder [29] and haddock in the regions further suggest that Atlantic cod [19] comprised the majority of early stage CHW eggs.

### Physical observations

Temperature can be used to estimate egg stage duration [30], [31], and was therefore the key linking variable in modelling scenarios for biological field observations. Continuous temperature data for Smith Sound were derived from temperature loggers secured at depths ranging from 10 – 40 m collected during the spring and summer from 2004–2007 (Fig. 1c). Vertical CTD casts for conductivity, temperature, fluorescence and depth (SeaBird Electronics SBE 19) were collected with each ichthyoplankton tow during the Trinity Bay Tucker trawl surveys, providing physical profile data for Smith Sound and Trinity Bay. We collected current measurements in Smith Sound, in 2007, with a 300 khZ RDI Acoustic Doppler Current Profiler (ADCP), deployed in an upward-looking configuration in the middle of the Sound (Fig. 1c).

We calculated the residence time for particles in the surface waters of Trinity Bay using a regional ocean circulation model designed for simulating ocean currents [22]. The model was run at 1 km resolution and forced with wind speed data collected at St. John's airport. The circulation field was determined for the summer period (June to September) for each year from 1953 to 1992. Particles (n = 49) were randomly placed in 3 km by 3 km boxes, 400 in total for Trinity Bay. Particles were tracked until more than half had left the bay and this duration was identified as the residence time for the particles in that box.

### Data analysis

Spatial patterns of egg and larval concentrations, and complementary biological/physical variables were interpolated between sample stations using kriging (Surfer® 9). Spatial estimates of egg and larval concentrations were obtained using kriging data interpolated from all stations sampled, thus providing a mechanism to map spatial heterogeneity. Based on preliminary examination of the data, which suggested an east-west discontinuity, we separated subsequent analyses by dividing the interpolated field into eastern and western groupings (Fig. 1b).

From the interpolated fields, the “centre of mass” (COM) of egg stage within a particular survey was calculated according to the equation:
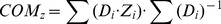
(1)


where *COM_z_* is the calculated centre of mass along either the latitudinal or longitudinal axis for a given latitude or longitude Z_i_, for station *i*, and *D_i_* is the observed concentration at the *i^th^* station. The interpolated output from linear kriging analyses was used as a mechanism to avoid, or at least minimize, spatial bias associated with closely-spaced stations (Fig. 1*b*). Passive transport rates were estimated from the distance between COM, calculated using great-circle distance and the transition time between early (1–2) and late (3–4) egg stages. Transition times were calculated according to temperature data from CTD measurements, utilizing development equations from Bradbury et al. [8]. To account for variability in the estimate of the mean temperature, and therefore passive pelagic duration, 1000 randomizations were run in Matlab using the maximum and minimum observed temperatures as constraints. Randomized mean temperatures were then used to calculate possible transport distances for the different egg durations. These error estimates enable a realistic comparison of transport distance variability based on real data and are independent of any specific statistical error structure. Estimates from this analysis provide a metric of diffusion of eggs within the Trinity Bay system.

Egg number as a function of distance from the presumed source was determined by multiplying the concentration of eggs observed by the mixed-layer depth (from CTD casts) and the radial surface area assigned to the station. The radial surface area was derived from the surface area of an arc sector radiating out from a vertex at the mouth of Smith Sound and expanding to the north-west and south-east boundaries of the sample array. The distance of a station from Smith Sound was determined from the great-circle calculation.

### Modelling

**Figure 2:**
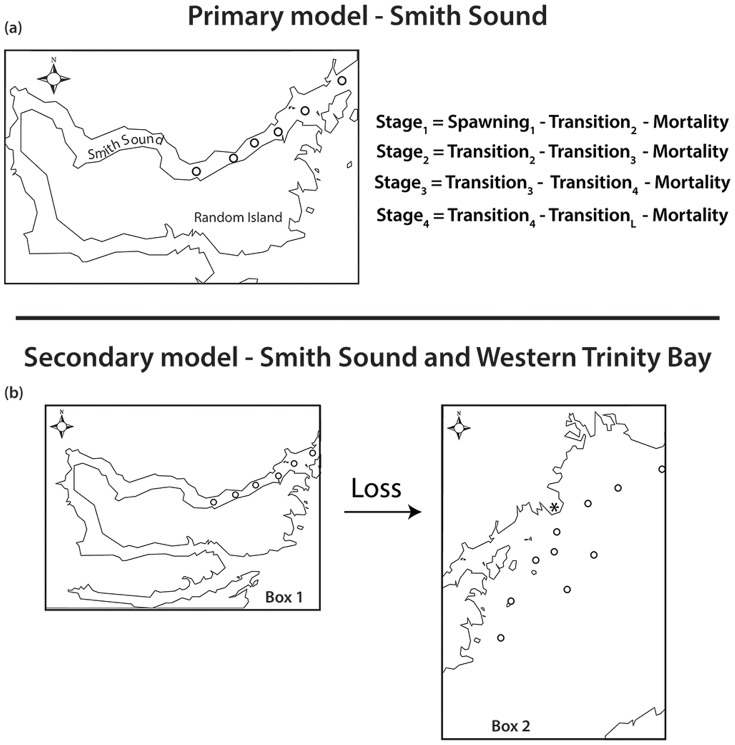
Illustration of model setup for primary (a) and secondary (b) models. Equations define daily egg numbers. Source terms of secondary model (**b**) are loss terms from primary model (**a**). “*” refers to Bonaventure Head.

**Figure 3:**
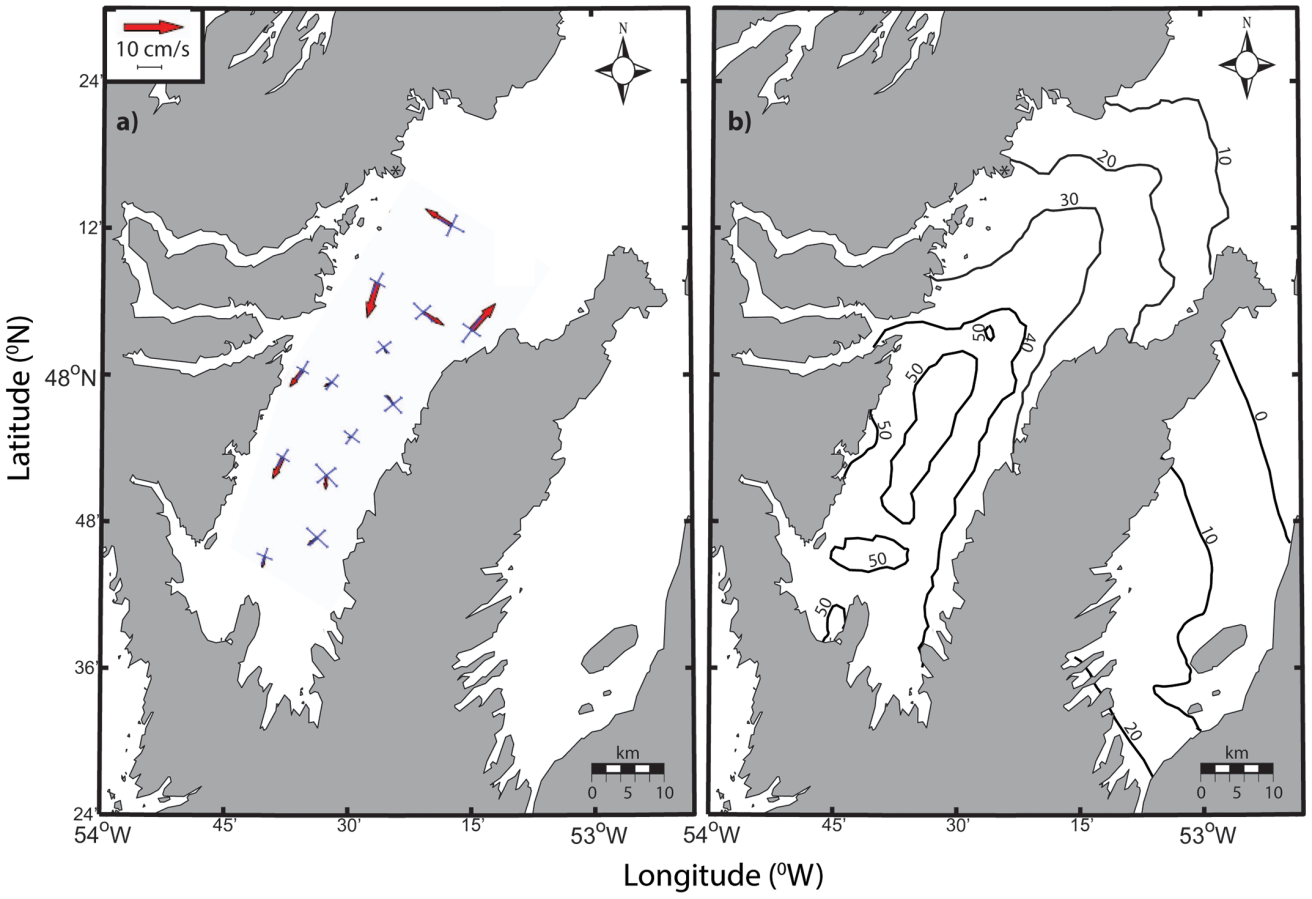
Results of regional circulation model detailing (a) average residence times for particles released in the surface waters of Trinity Bay (June-September, 1953–1992) and (b) mean currents in Trinity Bay (May-August 2002) at a depth of 40 m. The solid axes represent standard deviation of the flow along the direction of maximum and minimum variance. Redrawn with permission from Tittensor et al. [23].

A two-box model was coded in Matlab to estimate the parameters defining spawning and dispersal from Smith Sound. The primary box represents Smith Sound (Fig. 2a) where eggs are released, experience mortality, and progress through egg stages with each time step (daily). In the model, the box is a point, representing the area of Smith Sound. In the primary box, mortality is the combined effect of loss from death and loss from advection out of the system because eggs can leave the box at the end that connects to Trinity Bay. For each day, the model output dictates the numbers of each egg stage present in the Sound. The secondary box, which represents Trinity Bay, includes an input of eggs from Smith Sound to Trinity Bay (Fig. 2b). The eggs that exit Smith Sound are an output of the primary model, and enter as an input to the Trinity Bay box of the model. Thus the two boxes of the model are connected. We assume that the eggs leave Smith Sound and enter Trinity Bay but in essence the model representation is for the net result of transfer between the two systems and could be considered as the sum, at any particular time, of the eggs leaving Smith Sound and those entering the Sound. The model depends on the time-scales for this problem. The typical advective time-scale for Smith Sound is likely to be long relative to the model, since the mean currents in the Sound are weak, less than a few cm/s, leading to a time scale of roughly 20,000 m/10^−2^ m•s^−1^or, roughly 20 days. As Figure 3a shows the time-scale for residence in Trinity Bay is also quite long, 20–50 days, and so on the time scale of the model, a day, the system can be thought of as relatively stationary since the time scales in both systems is at least twenty times longer. These advective time scales are also long relative to the time that it takes to conduct a survey: 1–2 days for Smith Sound and 2–3 days for the Trinity Bay region. The times are also long relative to the transition times for the eggs, which are ∼ 4–5 days. The advective time scales of Smith Sound and Trinity Bay support the relevance of the model.

The primary model was constructed in Matlab using a three-vector Leslie matrix:
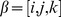
(2)


where *β* is the matrix representing Smith Sound, *i* is the vector tracking the cohort released over the spawning period (144 days), *j* is the vector that tracks day 1–144 and *k* tracks egg stage (stages 1–4 and larvae). Eggs are released each day according to the spawning scenarios described below. Each day (*j_i_* ) eggs progress through part of an egg stage (*I*–*IV*) according to the cumulative egg development durations (*D*) successfully employed to describe cod dispersal in neighboring Placentia bay by Bradbury et al. [8]. 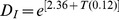
(3)

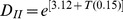
(4)

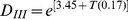
(5)

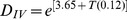
(6)


Each time step is associated with a temperature observation (T) from the Sound on the *j^th^* day. Each day, *j_i_*, the cohort completes a percentage of a stage (*k*) according to temperature development equations defined in Bradbury et al. [8] compiled from data presented in Pepin et al. [32]. Once a cohort completes 100% of a stage, it is lost from the preceding stage it then moves on to the subsequent stage on the next day ***j***_i+1_. Eggs are tracked and experience mortality collectively as cohorts. Daily cohorts include the previous day's cohort minus mortality loss for that stage. Mortality in the primary model is a single term varied from 0–99% which is a sum of death due to natural mortality and dispersion from the system. Essentially dispersion out of the box, at the mouth, is equivalent in the single box model to natural mortality.

The temporal characteristics of spawning were modelled using Gaussian- distributed spawning scenarios, varying in degrees of protraction, and an empirically-derived spawning model based on observed temporal patterns in egg abundance from the Smith Sound surveys (Fig. 1c). The peak egg abundance predicted by the Gaussian curves was set to coincide with the peak mean abundance observed in the Smith Sound surveys and to encompass the entire observed spawning period (March 30^th^ to August 20^th^). The primary model was tested with different spawning distributions (n = 6) and mortality levels (0–99%) to estimate both the temporal characteristics of spawning and net mortality in the system. The output from each model run consisted of a distribution of egg stage counts, with associated percent stage development, for every day of the spawning season.

The output of the egg time series from the primary model was compared to abundances estimated from bi-weekly Smith Sound surveys. The model tracks the relative abundance of eggs although it does not represent a concentration abundance that can be compared with the observations from the field. Instead of absolute egg numbers or concentrations, the model output and field abundances were compiled into relative frequencies of each egg stage (*k*) for given day (*j_i_*). In essence, the model and the observations are really relative indices of abundance rather than estimates of absolute abundance. The survey data and model prediction of relative frequency for each egg stage over the spawning period were compared, resulting in a coefficient of determination (r^2^) or least squares fit. For every model permutation, we varied spawning scenario (n = 6) and mortality values (0–99%) and calculated an r^2^ for each stage. The fit of the model permutation was evaluated by calculating the mean r^2^ minus the variance in r^2^, among all egg stages. This method maximized the model fit to all egg stages, and avoided erroneous parameter estimates associated with strong fits to some stages and weak fits to others. To account for variability in ichthyoplankton survey data (6 stations, Fig. 1c), a randomization was employed, constrained by mean egg stage concentration±1 standard deviation, to compute 1000 mean relative abundances of each egg stage, each sample day, and thus 1000 estimates of model fitness for each model permutation. Global model fits to each permutation, accounting for this ichthyoplankton variability, could then be assigned using the same algorithm described above (mean-variance). The spawning treatment and corresponding net mortality with the highest global model fit represented the best estimation of Smith Sound ichthyoplankton data.

Net mortality estimated from the model is the sum of both advective loss from the system and natural mortality (death, predation, etc.). To estimate advective loss from net mortality an estimate of natural morality was calculated from Trinity Bay ichthyoplankton data according to the equation: 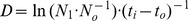
(7)


where *N*_o_ and *N*_1_ are the combined mean survey abundances for consecutive stages and *t*_o_ and *t*_1_ are the predicted egg stage durations. To account for the variability in abundance among Trinity Bay stations, abundance and temperature data from each survey were bootstrapped 1000 times to estimate a mean mortality rate and the associated standard deviation. Previous natural mortality rate estimates range from 0.1/day to 0.3/day [8], [33]–[37]. By estimating a possible range of natural mortalities observed in the field, the advective loss from the system given a known net mortality can be inferred.

The primary model estimates the spawning characteristics of the Smith Sound aggregation defining production and mortality. Mortality in the primary model represents the sum of egg mortality and advection from the system but cannot differentiate between the two. Egg stage abundance data collected from surveys in adjacent Smith Sound were utilized to estimate the advection from the primary model. In this case the advective component of mortality in the primary model was treated as the input into the secondary model representing Trinity Bay (Fig. 2b). The primary model scenario with the best fit was used to simulate egg production throughout the spawning season in Smith Sound. Again, as in the primary model, daily relative frequencies were used as indices of egg stage abundance because data did not permit parameter estimation using egg number. Each day the primary model predicts egg stage abundances which are multiplied by a dispersal term to be used as the source for eggs in the Trinity Bay system. The dispersal term varied from 1% to the net mortality estimated by the primary model. Each model permutation then provides egg stage abundances that are released into Trinity Bay, which then progress in daily time steps just as in the primary model. Loss rates were calculated by minimizing the sum of the squared differences between the relative frequency of each egg stage predicted by the model and observed in the field for each sampling day in each permutation. Data from 2004 provided enough temporal coverage (May and July) to fit a seasonal estimate of advection. The predicted relative abundance of each egg stage was compared to the field estimates taken on respective sampling days. As in the primary model, each permutation produced an r^2^ for each egg stage. The model fit was calculated by taking the mean r^2^ minus the variance in predicted r^2^ among stages. The randomization and global model fit procedure used in the primary analysis to incorporate variability in the field data was repeated with the western Trinity Bay data (10 stations; Fig. 1c).

## Results

### Particle tracking and residence time

In general, Smith Sound mean currents are less than a few cm/s and decrease in speed from the mouth to the head of the inlet. Current meter observations from Trinity Bay show that the mean flow speed is typically close to the variance [23] indicating substantial variability in the circulation relative to the mean flow pattern. Nonetheless, on average, water clearly enters on the west and exits on the eastern coast generating a weak anti-cyclonic flow. Current variability, primarily driven by wind stress [22], produces complex patterns of response dominated by an upwelling-downwelling cycle and a Kelvin wave signature [22]. A counter clockwise gyre observed near the mouth of Smith Sound exhibits some strong flow (Fig. 3b). Tracking models suggest residence times ranging from 20–30 days for particles released near the mouth of Smith Sound. As expected, much longer residence times near the head of Trinity Bay (50–60 days) greatly exceed those near the mouth (5–10 days). Overall, expected residence times for particles retained within the sampling array (Fig. 1b) ranged from 20 to 50 days (Fig. 2a).

### Field observations

**Figure 4:**
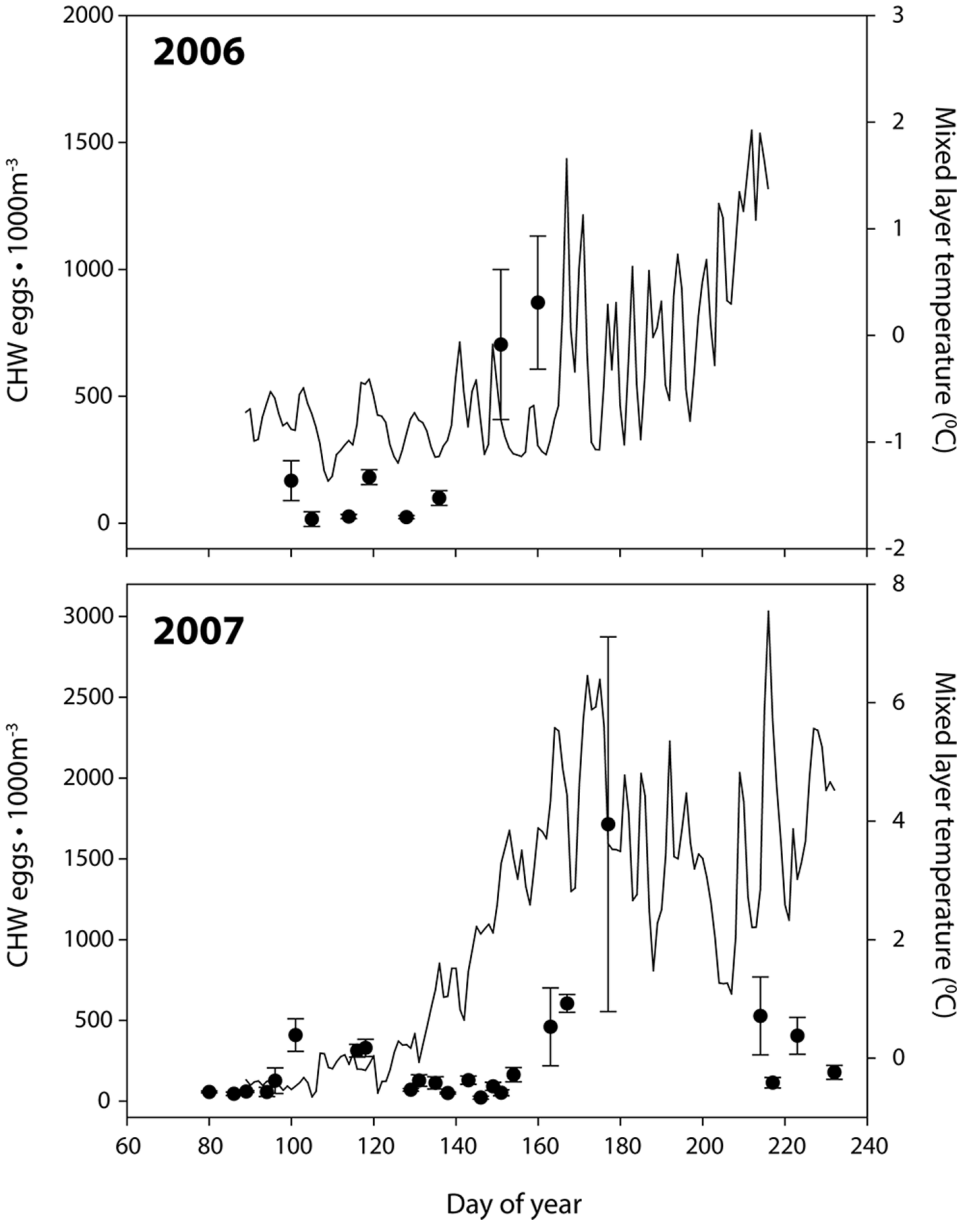
Mean stage 1 CHW egg density±standard error sampled in Smith Sound during 2006–2007 ring net surveys. Solid line indicates mean mixed-layer temperature (<40 m).

Mean concentrations of stage 1 CHW eggs were highest in Smith Sound during early summer (particularly early July 2007). Increases in egg concentrations through June and July also coincided with increases in mixed-layer temperatures (Fig. 4). Abundances of stage 1 CHW eggs, relative to all other stages, in Smith Sound correlated negatively with mixed-layer temperature among sample years (Pearson correlation = −0.368, p = 0.041, n = 29; controlled for year). All late stage CHW eggs were identified as Atlantic cod as in previous ichthyoplankton studies in Trinity Bay [15], [16] and nearby Placentia Bay [8]. All CHW eggs were therefore considered to be Atlantic cod with an unknown, but likely small, number of early stage eggs possibly misidentified.

**Figure 5:**
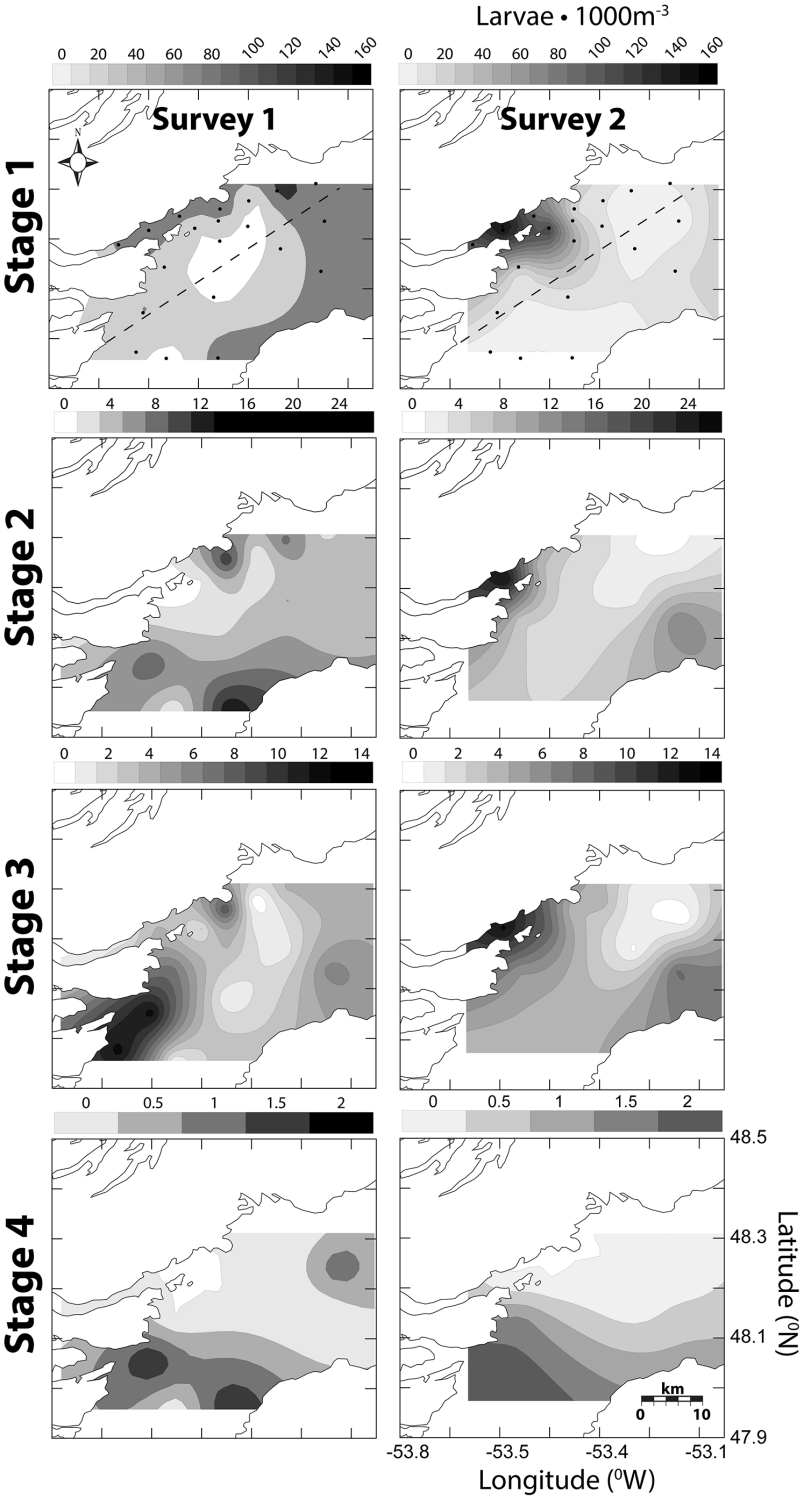
Spatially-contoured CHW eggs stage concentration (eggs•m^−3^) for May 2004 Trinity Bay Tucker trawl surveys. Dots represent station array and dashed line represents east west division.

**Figure 6:**
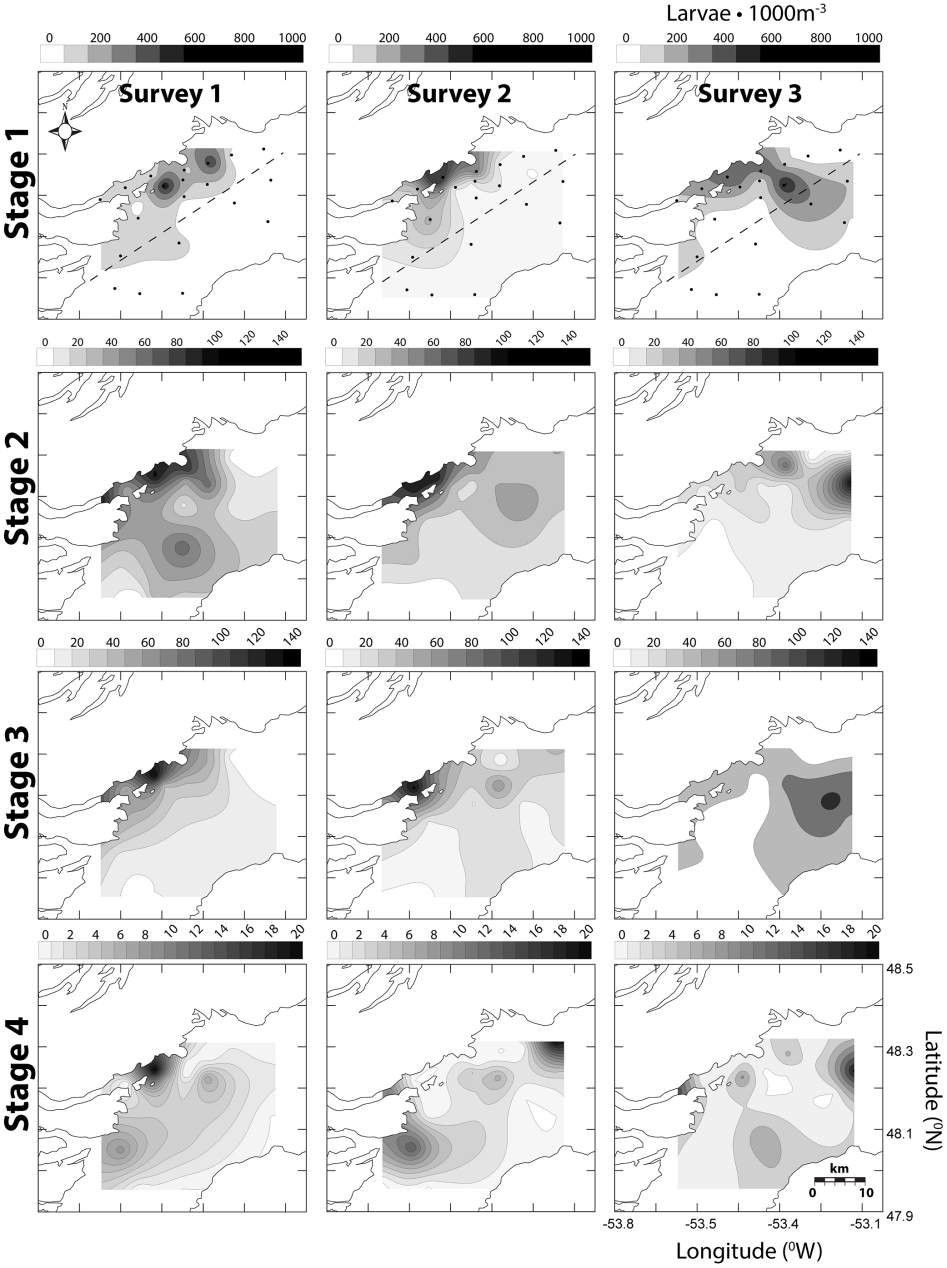
Spatially-contoured CHW egg stage concentration (eggs•m^−3^) for July 2004 Trinity Bay Tucker trawl surveys. Dots represent station array and dashed line represents east-west division.

**Figure 7:**
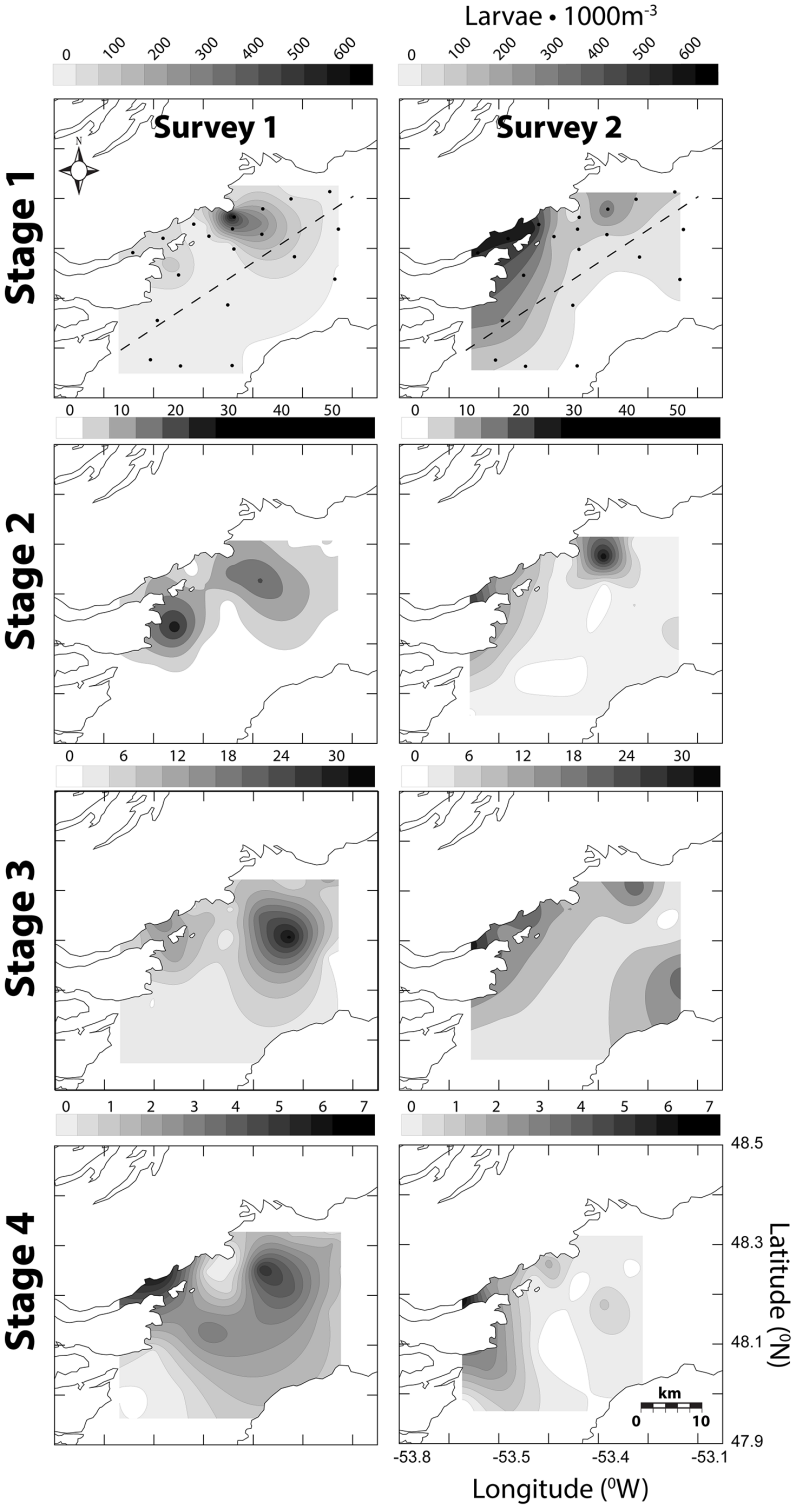
Spatially contoured CHW egg stage concentration (eggs•m^−3^) for May 2006 Tucker trawl surveys. Dots represent station array and dashed line represents east-west division.

The highest concentrations of early CHW egg stages (1–2) were closely associated with Smith Sound, the presumed natal source. Late stage CHW eggs (3–4) were typically most abundant on the western inner portion of Trinity Bay, as predicted from mean southerly transport of eggs flushed from Smith Sound. Spatial associations of stage 1 eggs were consistent among all surveys whereas late stage distributions, particularly stage 4, were much more variable (see Figs 5, 6, 7 for May 2004, July 2004 and May 2006 respectively). Egg concentrations from the July 2004 survey were the highest observed among all Trinity Bay Tucker trawl surveys; this timing was consistent with temporal spawning data from Smith Sound. In all surveys, the western side of Trinity Bay had significantly greater concentrations of egg stages (GLM egg density, side of bay, controlling for survey, *F* = 34.743, 16.306, 3.433, 84.490; p =  <0.0001, <0.0001, 0.064, <0.0001, for stages 1–4 respectively). Larvae were also significantly more abundant on the western side of Trinity Bay [7].

Area-corrected egg numbers (*see* Methods section) were unrelated to distance from Smith Sound. Regression analysis demonstrated that the egg number was effectively constant (slope  = 0.021, f = 11.048, r^2^ = 0.062, p = 0.001) as a function of distance from Smith Sound for all surveys and egg stages. There was also no significant trend in distance from Smith Sound and variance in egg concentrations (f = 0.225, p = 0.636) for individual or pooled surveys.

**Figure 8:**
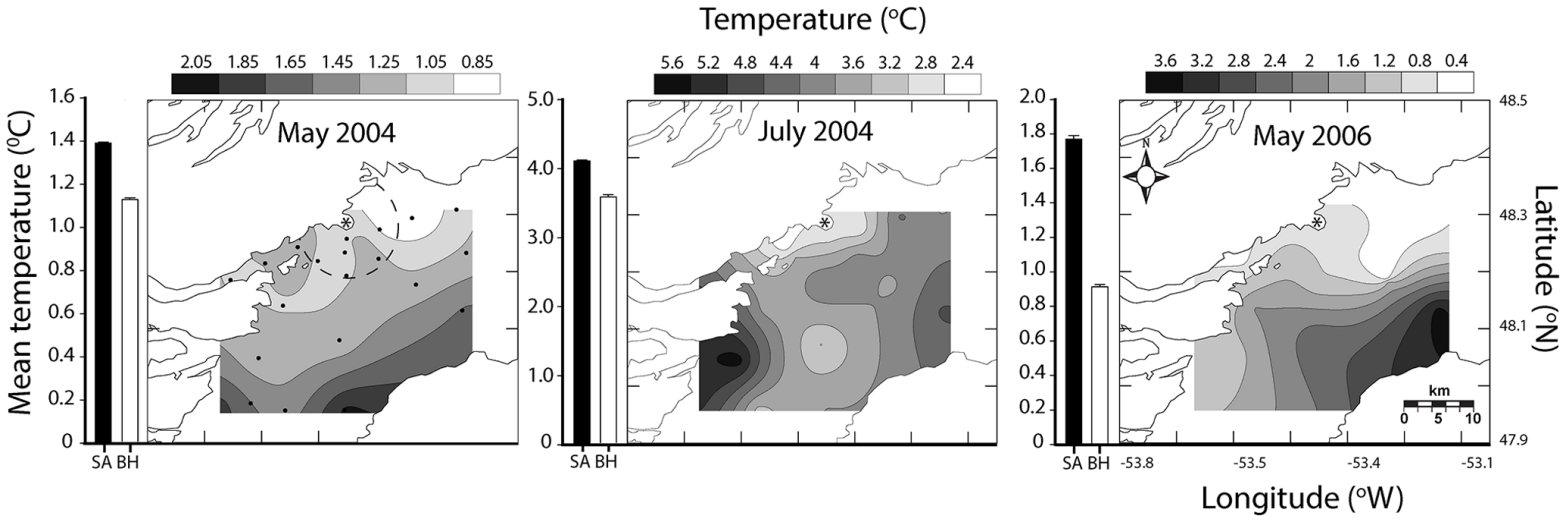
Spatially-contoured CTD cast temperature data for the mixed-layer (average up to 40 m depth) collected during ichthyoplankton surveys of Trinity Bay 2004–2006. Bar plots represent mean temperatures±standard error for the stations within (white bar) and outside (black bar) the upwelling zone (those within 10 km of Bonaventure Head). Dashed line represents the radius of temperature data sampled for Bonaventure Head (*) and dots represent station array used for all surveys.

Mixed-layer temperatures near Bonaventure Head (those stations within 10km), western Trinity Bay, were significantly colder than temperatures observed in the remaining stations (Fig. 8). This observation was consistent during all of the sampling periods in spring and summer (Table 1), and with previous studies in the area [20]–[23], [38]. Similarly, stations within the upwelling region were characterised by significantly higher primary productivity levels (fluorescence) and zooplankton abundance than remaining stations (Table 1).

**Table 1:** Results for General Linear Model Analysis of Variance comparing physical and biological parameters between stations within (those within 10 km of Bonaventure Head) and outside the upwelling zone.

Variable	Factor	df	F-value	p-value
Fluorescence	Upwelling zone	2	5.836	0.0037
	Survey	6	35.037	<0.001
	Interaction	7	0.939	0.4788
				
Mixed-Layer Temperature	Upwelling zone	2	3.701	0.0272
	Survey	6	35.037	<0.001
	Interaction	7	0.166	0.9914
Zooplankton*	Upwelling zone	1	6.377	0.0162
	Survey	1	3.925	0.0501
	Interaction	1	0.957	0.3347

* zooplankton data only available for 2006 surveys

Centre of mass (COM) calculations conform to predictions of relative differences between early (1–2) and late (3–4) CHW egg stages based on temperature derived duration. The longest observed distance between early to late COM was the May 2006 mean distance of 7.6 km (standard deviation (s.d.) = 0.3). Distances were less for July 2004 (2.8 km, s.d. = 0.5) and May 2004 (3.0 km, s.d. = 2.4) respectively. The shortest COM distance was in the first survey of July (0.2 km) but subsequent survey rounds during the same cruise produced a mean distance of 4.4 km (s.d. = 0.3). Based on known development rates (*see* Methods section) passive transport rates were estimated to be 0.43 km•day^−1^ (s.d. = 0.25) and 0.32 km•day^−1^ (s.d. = 0.18) for surface and mixed layer temperatures respectively. Centre of mass calculations tend to produce conservative transport distances because they artificially mask symmetrical bidirectional dispersion. However spatial observations (Figs 5, 6, 7) coupled with COM locations suggest a reasonable approximation of mean transport conditions in the field.

### Model results

**Figure 9:**
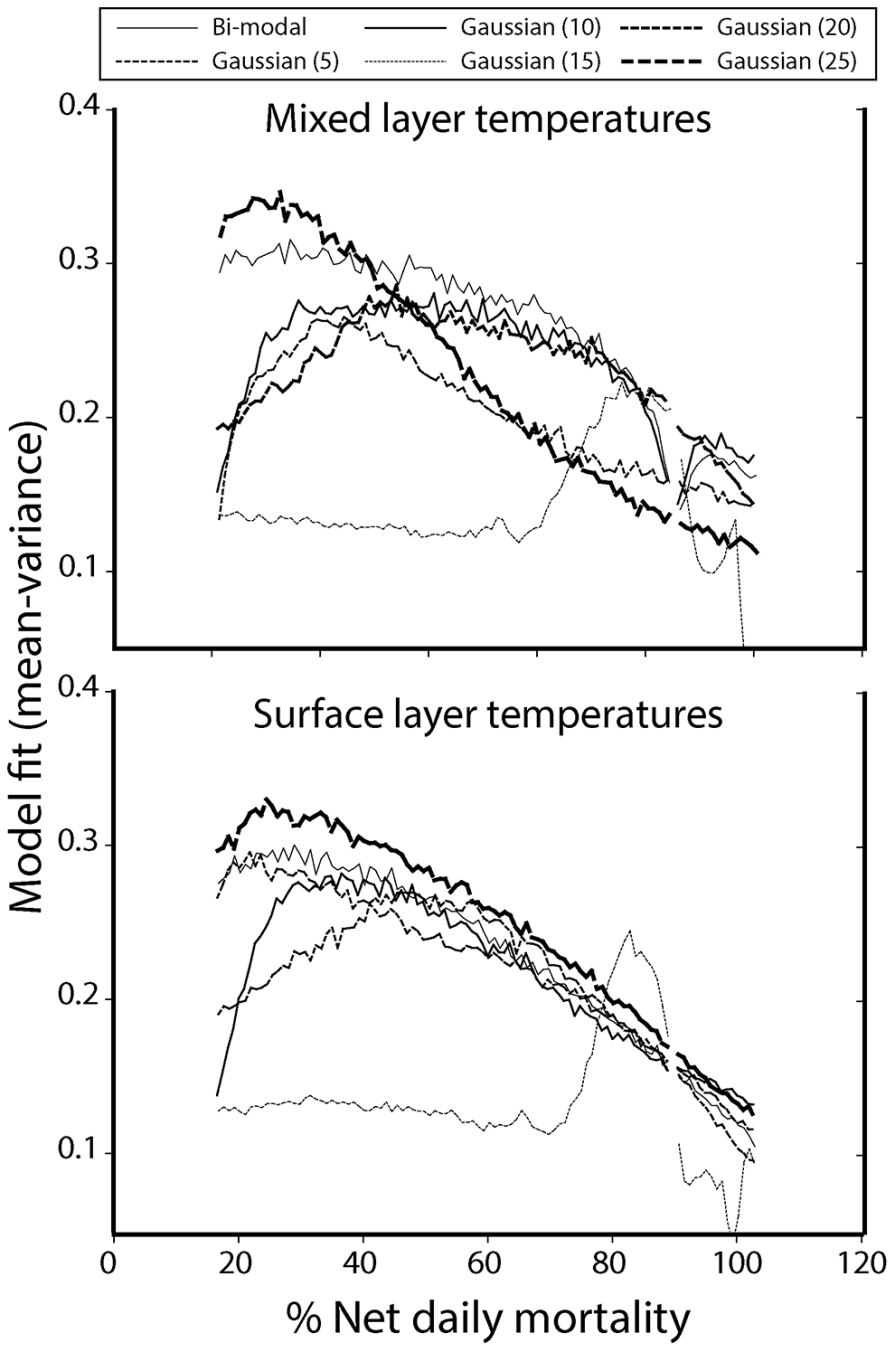
Model fit results for varying spawning scenarios, temperatures and estimated daily net mortality. Data presented show the overall mean of the model fits produced in 1000 randomizations of Smith Sound data.

Model simulations indicated that the most protracted spawning scenarios produced the best fit (mean - variance) for the 1000 randomizations of survey data for both mixed-layer and surface-layer temperatures (Fig. 9), with mixed layer temperatures yielding the best predictive capacity at 73% explained variance. The weakest predictive outcome for the temperature treatments (surface and mixed-layer) was the empirically approximated bi-modal distribution. Gaussian distributions and mixed-layer temperatures were therefore used for the remainder of the modelling analyses.

**Figure 10:**
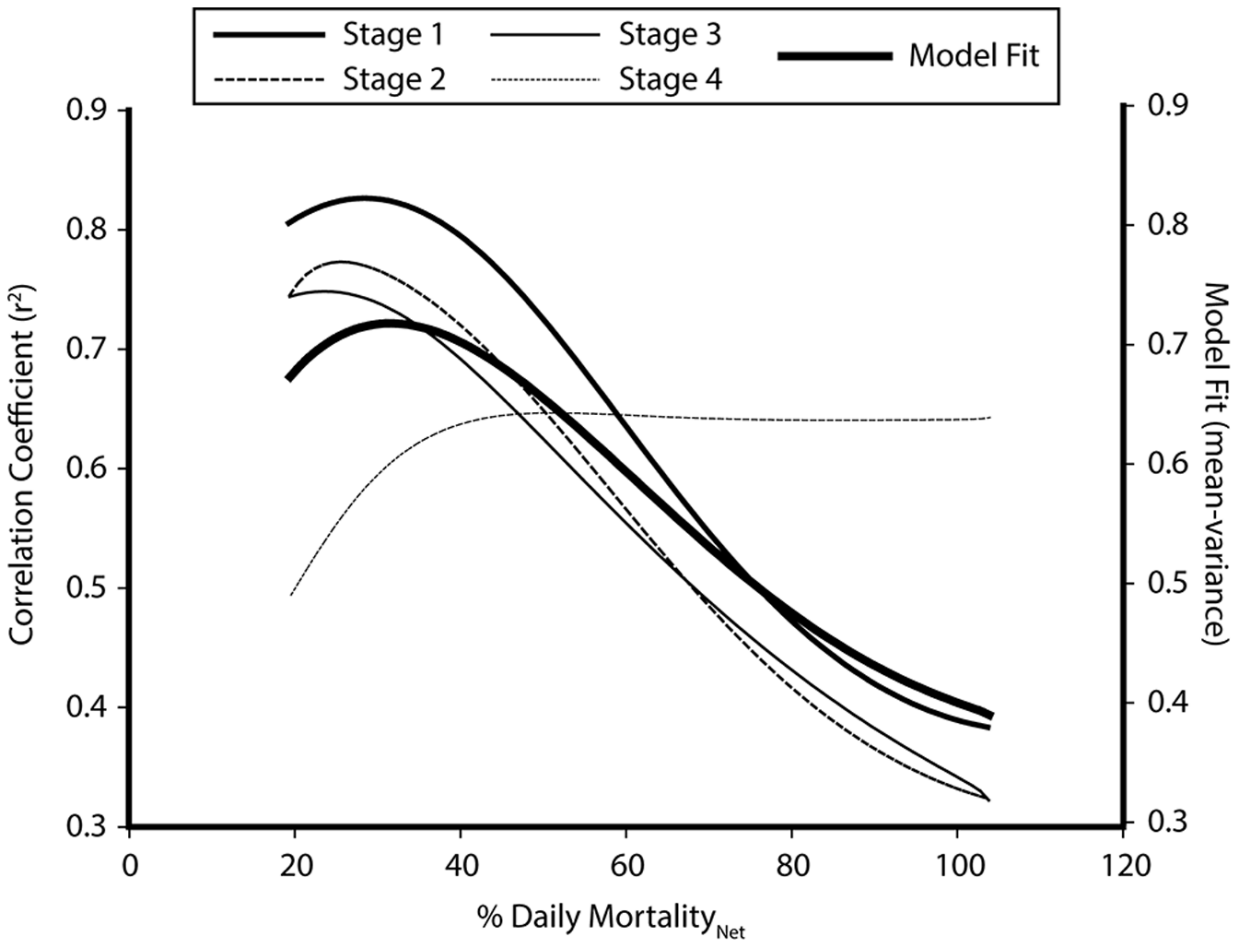
Model results for explained variance (r^2^) for each egg stage with varying net mortality. Also plotted is the overall model fit (mean minus variance in r^2^ among stages).

Daily natural mortality (death) was estimated using field egg abundance data at ∼7% (s.d. = 1%). In contrast with the mortality estimate above, models compared with Smith Sound ring net survey data estimated net mortality at approximately 27%•day^−1^, explaining approximately 73% of the variance in the data among stages (Fig. 10). This estimate of net mortality corresponds to a daily loss rate from Smith Sound of ∼20% (Loss_ = _Net Mortality – Natural Mortality).

**Figure 11:**
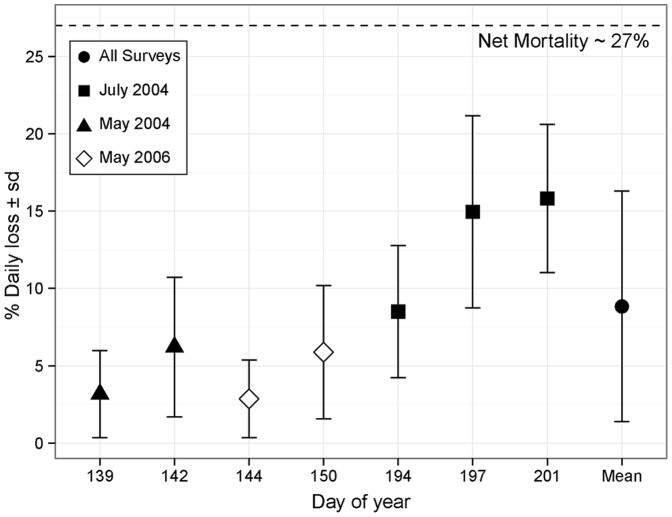
Estimates of percent daily advection of CHW eggs from Smith Sound (± 1 s.d.) during spring and early summer 2004–2006. Error bars are estimated from 1000 randomizations of western Trinity Bay survey data. Dashed line represents the estimated 27% net daily mortality from the Sound.

Loss rates estimated from western Trinity Bay data ranged from 2–15%•day^−1^ for individual surveys and were significantly positively correlated with day of year (GLM, f = 64.689, p<0.0001) (Fig. 11). The average loss rate estimated from all individual survey estimates was 10.3%•day^−1^ (s.d. = 7.7%). Standard deviation associated with mean estimates of loss demonstrated no significant trend with day of year (GLM, f = 4.616, p = 0.084). In 2004, there were enough sample days to estimate the parameters for among-stage model fitting that was utilized in the primary model. Daily loss rate from Smith Sound was estimated from the 2004 spawning season regression model at 13%•day^−1^, with higher variability (s.d.  = 11%) than that found for individual surveys. Based on all loss estimates derived from individual surveys and annual data, the mean loss rate from Smith Sound was estimated to be 9%•day^−1^ (s.d. = 7.5%).

## Discussion

Understanding movement and survival during pelagic dispersive stages of benthic organisms is critical to the evaluation of how populations are linked both genetically and demographically [3]. Inshore spawning of Atlantic cod has been well documented [39] and tied to both stock structure and population stability [40]. Central to the study of inshore spawning has been the evaluation of biophysical interactions which define placement of larvae to suitable settlement habitat [41] or retention within the inshore nursery environment [8], [42]. Our study offers the first empirically-derived measurements of early life history dispersal, and associated environmental factors, from a *discrete source* of Atlantic cod eggs and larvae, and thus represents a model system and analytical approach for inferring dispersal of pelagic spawners in coastal embayments. Using this approach we were able to generate mortality and dispersal estimates to describe dispersal in an inshore system.

After a nearly two decade-long fishing moratorium and no clear signs of recovery, the potential to forecast the future of the northern cod stock depends on understanding its current spatial structure and mechanisms of connectivity. The cod in Smith Sound, discovered in 1995, form the largest known spawning aggregation in the northern cod stock complex. Despite recent indications that the Smith Sound stock has reduced substantially in size [12], its anomalously high abundance and patchy nature have attracted significant research attention, including monitoring of Smith Sound annual adult biomass [43]–[45] behavioural studies [18], [44], [46], [47], measurements on physiological traits [17] and speculation on the spatial and temporal origins of the stock [12]. As is common for other harvested fish populations worldwide, however, little detailed information exists on dispersal and connectivity of egg and larval stages so important in determining overall population viability. In the case of Smith Sound, the role of dispersal of progeny from the area has received less empirical attention than for other offshore stocks [48] and nearby Placentia and Conception Bays [49], [50].

Observations from Smith Sound ichthyoplankton surveys in 2006 and 2007 indicate a bi-modal, protracted spawning pattern, from March to August, peaking weakly in late May and strongly in mid to late-July, confirming a complementary study by Knickle and Rose [19]. Peak egg production in late July coincided with increases in temperature, exposing these eggs and their development to the highest expected annual temperatures (July and August). Model simulations are consistent with strong temperature dependency and protracted, rather than discrete, spawning events. Models incorporating the protracted spawning scenario provided the best fit to field data. In this respect, our results were consistent with previous studies that documented protracted spawning in Atlantic cod [51] and inferred timing of Trinity Bay cod spawning from ichthyoplankton surveys [46]. Both modelling and field data show a strong positive relationship between stage 1 CHW egg abundance and temperature (*see* Fig. 4). The observed temperature dependence builds on previous results that suggest environmentally-driven spawning in Smith Sound [46] and nearby Conception Bay [50] strongly related to temperature [49]. Other studies report regional variation in timing of cod spawning [52], [53], and link spawning variability to temperature and other regional differences.

The protracted nature of cod spawning, and seasonal changes in environmental condition, lead to the prediction that seasonal variation in spawning influences annual recruitment success [8]. Temperature can drive pelagic egg mortality both negatively [54] and positively [30], [31], depending on the underlying process (i.e. growth or predation). In particular, spawning in colder temperatures extends pelagic durations thus prolonging exposure to high mortality rates characteristic of pelagic stages [54] and increasing the potential for dispersal from inshore nursery areas [8], [55]. Data comparing early spawning (May) to mid-summer (July) offers an alternative to this generalization. The ratio of peak stage 1 to stage 4 egg abundances did not vary seasonally, indicating that survival of the egg stage likely does not vary significantly among seasons despite a 1-week extension in development times early in the season (based on mean mixed-layer temperatures). The consistency in spatial patterns seasonally suggests that oceanographic processes can facilitate retention and persist through the spawning period.

Spawning at warmer temperatures results in faster development and reduced pelagic durations, thus leading to generally shorter passive transport distances and increased local retention [4], [7], [8]. Our results generally confirm this pattern. Specifically, the longest transport distances estimated among all surveys occurred when the coldest temperatures were recorded. These results indicate that the timing of Smith Sound spawning appears to favour retention, which the presence of late eggs stages and larvae in and near Smith Sound confirm. The majority of spawning activity occurs at times that decrease pelagic egg durations and therefore increase retention in and around Smith Sound.

Oceanographic conditions in Trinity Bay may enhance the retention of eggs and larvae near the sound. Upwelling and other oceanographic features, such as gyres, have been shown to impact spatial patterns of pelagic propagules of a variety of species including Atlantic cod [56]. In Trinity Bay, persistent upwelling [20], [23], [38] and a gyre spanning the width of the bay [21], [23], near the mouth of Smith Sound, may influence dispersal trajectories of propagules originating from the sound as suggested by Dalley et al. [55] for larval capelin (*Mallotus villosus*). Large-scale modelling of Atlantic cod dispersal on the Norwegian coast demonstrated similar patterns, where coastal retention of eggs and larvae was attributed to small-scale and localized eddies found between islands along the coast [57]. Our data demonstrated a general association of all stages of eggs and larvae with the western side of Trinity Bay. The transition time from stage 1 to stage 4 is 26–33 days based on observed mixed layer temperatures (equations 3–6) [8]. Mean passive flow conditions estimated from flow modelling [20], [21], ADCP data [23], and particle tracking (Fig. 3a) could move eggs beyond the sampling spatial window in as few as 20 days, based on observed mean flow estimates of roughly 10 cm•s^−1^and Euclidean movement. Passive flow rates inferred from ichthyoplankton concentrations are 0.32 km•day^−1^ for mixed-layer temperatures and 0.43 km•day^−1^ for surface temperatures represent less than 5% of estimated mean flow in Trinity Bay, further suggesting a strong role for the upwelling and gyre features. Even if the estimation of ∼5% of expected flow rates is extremely conservative, the concurrent observation of spatial patterns is inconsistent with dispersal at the mean current rates estimated in the area. In addition, no consistent relationship between egg numbers and distance from Smith Sound was observed, despite an expected decrease in number as a result of diffusion and cumulative mortality from the expected source. This result supports our conclusion that spawning is protracted and dispersion and mixing of eggs from the Sound. Spatially, stage 1 eggs were consistently associated with the assumed natal source, Smith Sound, whereas stage 4 eggs varied among surveys, further negating a simple Euclidian dispersal interpretation.

Ichthyoplankton data collected in this study show that Smith Sound represents the major source of CHW eggs in the Trinity Bay system. The highest abundance of stage 1 CHW eggs is consistently associated with the western side of Trinity Bay near the mouth of Smith Sound from spring through summer and from year to year (Figs 5, 6, 7). Only during one survey in mid-July 2004 was there any significant progression of egg stage concentrations towards outer stations near the mouth of Trinity Bay (Fig. 6, survey 3). Given the size of the Smith Sound stock relative to potential neighboring inshore sources at the time of sampling, detecting any outside recruitment input would be difficult, because any signal would be drowned by output from Smith Sound. Spatial analysis alone clearly illustrates the importance of the Smith Sound spawning aggregation to the Trinity Bay system, but suggests little movement of early life history stages outside Trinity Bay relative to local retention.

Model simulations indicate a ∼27% daily net mortality in Smith Sound very close to the rate of 28% estimated for silver hake (*Merluccius bininearis*) on the Scotian shelf using a variational numerical modelling simulation [36]. Our model simulations indicate that daily advective loss from the Smith Sound system varies between 2–15% with a mean of 13% for 2004 and ∼9% for all surveys combined. These results also show an increase in advective loss with day of year. The seasonal variation in estimated loss rate might reflect differences in spawning output during the protracted spawning season. A four-fold increase in spawning (estimated from Smith Sound surveys) in July within a two to three week period would spike the relative frequency of stage 1 eggs, driving the seasonal model of loss rates. Trinity Bay ichthyoplankton data demonstrate an increase in the relative frequency of stage 1 eggs near the peak July spawning activity relative to May surveys, consistent with model predictions. The 13% daily loss estimate from 2004 represents the best estimate because it incorporates seasonal differences in spawning and temperature. This estimate of loss represents the first empirical calculation of output of progeny from a discrete inshore population component of Atlantic cod, namely Smith Sound, providing a foundation for determinations of source-sink dynamics relevant to the species.

### Summary

The data and model simulations presented suggest that timing of spawning in Atlantic cod acts in concert with oceanographic features to retain larvae locally where food conditions are generally favourable and may be detectable by spawning adults. Contrary to studies in other systems [8], there is little evidence for seasonal differences in propagule retention. Analysis of spatiotemporal patterns of early life history stages suggests that connectivity potential from spawning decreases with distance from the spawning source, Smith Sound. Daily loss estimates for Smith Sound range between 8–13% daily. Overall, our data suggest high retention of egg and larval stages within the Smith Sound-Trinity Bay system. Although the data to fully explore the effects of wind forcing and circulation are lacking, the parameters and observations presented in this study are consistent with, and build on, existing data on early life history dispersal and connectivity in Trinity Bay and elsewhere.

Acoustic tracking data from adults in spawning conditions suggests that cod undergo migrations from Smith Sound into Trinity Bay and adjacent large bays [12]. Recent analysis suggests that since 2009 a considerable portion of the Smith Sound stock may have dispersed to the Bonavista corridor [12]. Currently the contribution of this spawning behaviour to population connectivity remains unresolved. It has been proposed that inshore spawners may eventually contribute significantly to the recovery of offshore stocks [43], [58] after density-dependent inshore-offshore spillover occurs among adults. Not surprisingly, our observations of a highly retentive system appear to corroborate this suggestion but only minimally; further quantification is required before the full influence of inshore spawning on offshore populations can be determined. Connectivity from the early life history of Atlantic cod arising from Smith Sound, and potentially additional coastal analogues may be low, likely playing a significant structuring role only on small spatial scales (e.g., 10's of kilometres).
